# Can FDI facilitate green total factor productivity in China? Evidence from regional diversity

**DOI:** 10.1007/s11356-021-18059-0

**Published:** 2022-02-25

**Authors:** Jialu You, Hang Xiao

**Affiliations:** Institute of Finance & Economics, Shanghai University of Finance and Economics, 777 Guoding Rd, Shanghai, 200433 China

**Keywords:** Green total factor productivity, Regional diversity, Pollution paradise, Bottom line race, Environment policy

## Abstract

The development of foreign direct investment conforms to the theoretical principles of green total factor productivity and is key to promoting regional industry upgrading. Using three-stage data envelopment analysis (DEA) based on city-level data, this paper investigates the effect of foreign direct investment on regional green total factor productivity (GTFP) across China. The results show that foreign direct investment affects regional GTFP through technology spillover effect and human capital spillover effect. Under different environmental regulation intensity and marketization, the relationship between FDI and green total factor productivity is non-linear. The phenomena of “pollution paradise” and “bottom line race” survived at low marketization regional and foreign direct investment will inhibit the improvement of regional green total factor productivity in China.

## Introduction

**Fig1:**
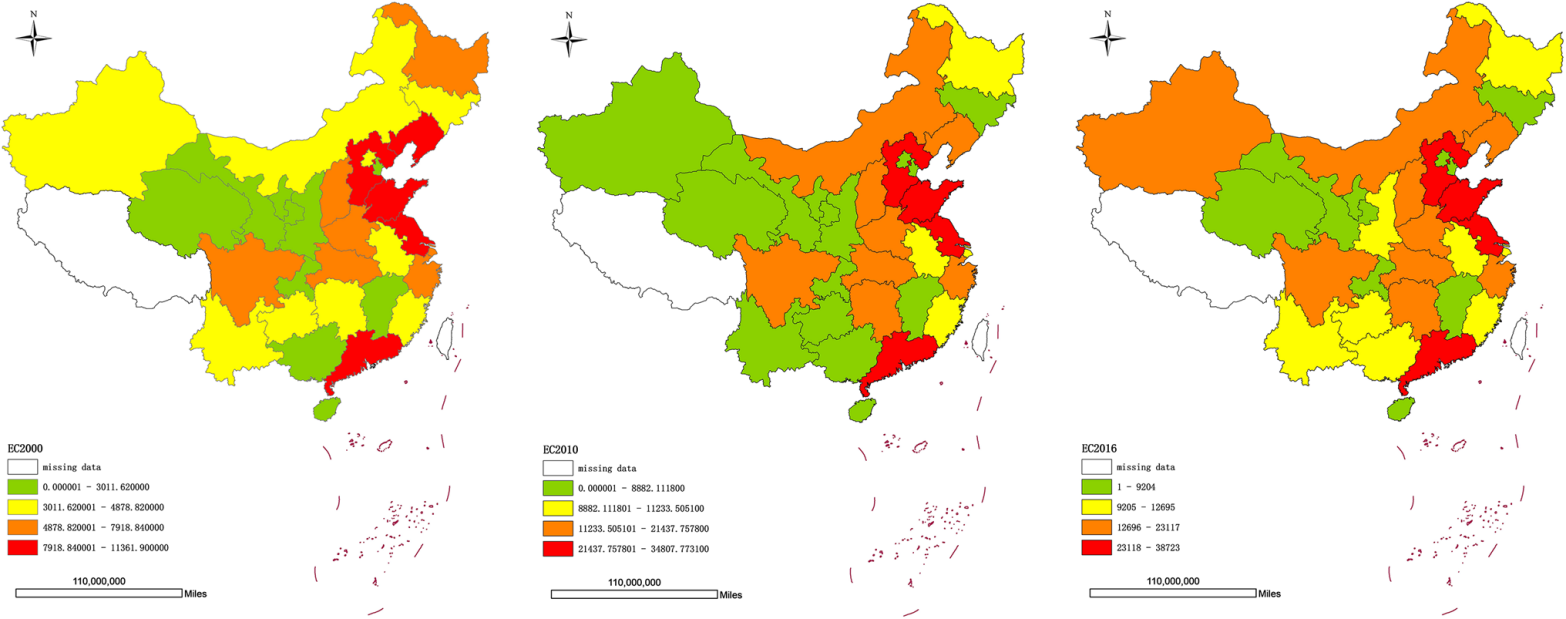
Spatial quartile maps of energy consumption (unit: 10,000 t of standard coal) in China from 2000 to 2016

The world economy is undergoing regional industry upgrading. As the largest energy consumer country, China’s energy consumption has increased year by year (see Fig. 1), accounting for 24% of global energy consumption and 34% of the global energy consumption growth.^1^ Motivated by the prevalence of economic targets at all levels of territory administration in China, local governments also tend to replace longer-term goals with short-term economic growth (Li et al. al. 2019). However, China’s economic development facing the growing pressure of resources and environmental constraints and sustained environmental degradation has negatively affected the quality of China’s economic growth, public health, and seriously hurt government`s reputation (Fu 2008). In response to the crisis of environment and energy, the Chinese government put forward the concept of “beautiful China,” emphasizing that the construction of ecological civilization should be placed in a prominent position, and pollution prevention should be included in the government’s key tasks.^2^ With the Chinese government attaching more importance to the environment and the relevant environmental laws and regulations are issued, enterprises will bear the rising costs of environmental protection, and foreign direct investment (FDI) flows to China will also be affected.

**Fig2:**
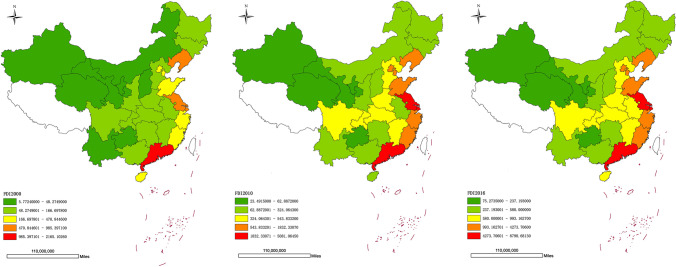
Spatial maps of foreign direct investment (unit: thousands of dollars) in China from 2000 to 2016

For developing countries, FDI is an important channel for undertaking international technology transfer, cultivating the competitiveness of local industries, and realizing capital accumulation, technology accumulation, and human capital accumulation. China’s rapid economic growth since the 1980s also benefited from the positive role of FDI in local industrial upgrading, technological innovation, and talent training (Kui-Yin and Lin 2004; Ullah et al. 2020). However, existing researches have not reached a consistent conclusion about the impact of FDI on the ecological environment of developing countries. There are two points about it: the “pollution haven hypothesis” (for example, see Reppelin, 1999; Ashford, et al. 2002; Eskeland and Harrison 2003; Abdouli, et al. 2017) and the “pollution halo” hypothesis (Birdsall et al. 1993; Abdouli et al. 2018). Kuznets (1955) proposed that the relationship between environmental pollution and economic growth was inverted U-shaped. In the early stages of economic development, developing countries face environmental degradation with multinational companies transferring pollute companies, which pursue the max profit and avoid the environmental cost, developing countries become “pollution paradise” (Eskeland and Harrison 2003). Pazienza (2019) proved that the impact of FDI on the intensity of carbon dioxide emissions is an inverted “U” shape in OECD countries. Series researchers show that “pollution paradise” hypothesis has been confirmed in China, increasing FDI which has a negative impact on the environment. Results from a top-down amplification of economic growth targets along with the jurisdiction levels; local governments tend to attract foreign investment with lower pollution costs (Luo et al. 2010; Chen et al. 2014). On the contrary, other researches proved the positive effect of FDI on the improvement of the host country’s environment. Both FDI and international trade provided impetus and opportunities for developing countries to develop advanced environmental protection technologies and improve environmental quality (Birdsall et al. 1993). Antweiler et al. (2001) showed that technological progress caused by trade reduced pollution in China and played a positive role in improving the environment. Karen et al. (2004) proved that technological effect of FDI in China had a significant positive impact on the environment by analyzing 2500 large and medium-sized industrial enterprises. Abdouli et al. (2018) verified the “pollution halo” effect of FDI in BRICS countries; once FDI reached the threshold level, it would have a positive impact on reducing carbon dioxide emissions. Singhania et al. (2021) researched the correlation between FDI, institutional, financial development, and sustainability in 21 developed and developing countries with high carbon emissions, and the results showed that FDI had a significant positive impact on the environment (Fig. 2).

Previous researches have noticed the effects of FDI on China’s economic development transformation and production efficiency, but the role of FDI in promoting green total factor productivity is not fully clear; the mechanisms of FDI on GTFP are still controversial. Due to different regional endowment and development levels, traditional efficiency measure methods, which ignore the influence of random factors and the external environment factors, may cause measurement errors to a certain extent and distort the analysis of the impact of FDI on GTFP. On the other hand, purely researching the regional heterogeneity of GTFP without considering a foreign investment, it is not conducive to discover the deep impact of China’s integration into the world market on its economic development transformation from the perspective of an open economy. This paper contributes to the existing literature in the following two aspects: First, after eliminating external environmental factors and random factors, we re-measure regional GTFP and reexamine the impact of FDI on the environment, further enriching the research on this field. Second, the objective existence of regional development differences leads to the heterogeneity of FDI’s environmental effects. Therefore, this paper uses panel data to analyze the mechanism of FDI’s effect on GTFP and its regional heterogeneity.

### Literature and hypothesis

FDI mainly affects the host country’s GTFP through three channels: technology spillover, innovation spillover, and human capital spillover. First is the technology spillover effects. On the one hand, FDI mostly enters China in the form of investment and plant construction. Since companies in the same industrial value chain need to use uniform standards and technical specifications, industrial linkages will force host country companies to adopt more advanced technologies. It also encourages local companies to increase productivity through “learning by doing” (Javorcik 2004), save factor inputs, and reduce industrial waste emissions. On the other hand, due to multinational enterprises’ needs to implement the environmental standards unified with the home country, it could promote the improvement of the host country’s environmental protection technology and effectively control pollution emissions (Eskeland and Harrison 2003). Second is the innovation spillover. After a multinational enterprise enters the host country, it will compete with local enterprises in the product market, production factor market, and human resource market to capture limited resources, intensifying the factor market competition (Magnus 1998). It encourages local enterprises to carry out technological research and development to improve competitiveness and improve the productivity of the host country. Third is the human capital spillover effect. By training local employees, transnational enterprises internalize human capital in local employees. With the flow of employees between enterprises, human capital spillover is helpful to improve the human capital of enterprises in host countries (Noorbakhsh et al. 2001). However, FDI inflow also has a certain “crowding out effect” on enterprises in the host country (Ari Kokko 2014). The impact of the above three effects depends on whether enterprises in the host country breakthrough the dependence path of introduction technology of FDI, forming a virtuous cycle of “introduction–absorption–innovation–output.” If the enterprises in the host country cannot absorb technology, carry out independent innovation, and form a high-quality labor pool, FDI will inhibit the improvement of GTFP in the host country. Therefore, this paper proposes hypotheses 1 and 2:

- H1: Technology spillover effect, innovation spillover effect, and human capital spillover effect brought by FDI will affect the GTFP of the host country.
- H2: The influence of FDI on GTFP is subject to regional development differences.

Whether FDI can promote the improvement of green productivity in host countries is influenced by factors such as the level of local environmental regulation and marketization (Alfaro 2004; Asiedu 2006). Because enterprises in developed countries are often constrained by strict environmental regulations of the home country, and put large investment in environmental protection and pollution treatment. In order to avoid environmental costs, enterprises in developed countries often choose to set up multinational companies and relocate high-polluting and energy-consuming industries to developing countries. In order to pursue economic growth, developing countries often set a low environmental threshold to obtain a “comparative advantage” and attract FDI. In this case, countries with laxer environmental regulation become “pollution paradise.” China pursues economic development, and its regional development is a prominent imbalance, so the foreign investment is essential to help the country achieve industrialization. In the past few decades before 2012, the requirement of FDI is not stringent, the objective existence of "Race to the Bottom" among local governments, FDI bring a considerable amount of high-pollution and energy-consuming plants. Therefore, the degree of marketization and environmental regulation level play a moderating role in the “FDI-GTFP” relationship, mainly reflected in the following aspects: first, strict environmental regulation inhibits the inflow of low-quality FDI and attracts clean FDI, which is conducive to the improvement of regional GTFP. Second, the higher degree of regional marketization level, the local government’s administrative intervention in economic development is smaller, which means the greater the competition between enterprises and factors flow more freely. It is advantageous to weed out low-quality FDI in the marketplace and also benefit local companies to absorb advanced management experiences and technology by frequent technical exchanges and staff turnover. Hypothesis 3 is proposed in this paper:

H3: The influence of FDI on GTFP is nonlinear under the different intensities of environmental regulation and marketization levels.

## Methodology and data

### Model and method of estimation

#### Three-stage data envelopment analysis

Existing research on China’s green total factor productivity mainly focuses on industry green total factor productivity (Zhu et al. 2018), regional green total factor productivity (Yangjun 2019; Wei-Bing et al. 2019; Liu et al. 2020; Xia 2020), and urban green total factor productivity (Liu and Xin 2019). And the models such as BBC-DEA, DEA-SBM, and DEA-Malmquist are mainly used for calculating GTFP. Considering previous studies often ignore the influence of external environmental factors and random factors, this paper will use the three-stage DEA (Fried 2002) to measure regional green total factor productivity. The main steps of the three-stage DEA are as follows:

In the first stage, use the traditional BBC-DEA model (proposed by Charnes et al. (1978)) to calculate efficiency. The model equation is as follows: there are *n* DMU and $\overline{n }$ SU; each SU has *m* inputs and *n* outputs. Let ${x}_{ip}$ and $\overline{{x }_{ij}}$, respectively, denote the ${i}_{th}$ input of the ${p}_{th}$ DMU and the ${j}_{th}$ SU, while ${y}_{rp}$ and $\overline{{y }_{rj}}$, respectively, denote the ${r}_{th}$ input of the ${p}_{th}$ DMU and the ${j}_{th}$ SU. Therefore, the BBC-DEA model of the ${p}_{th}$ decision-making unit is shown in Eq. (1)

1 $$\begin{array}{c}MIN\left[\theta -\varepsilon \left(\sum\limits_{j=1}^{m}{s}^{-}+\sum\limits_{j=1}^{s}{s}^{+}\right)\right]\end{array}$$

2 $$\begin{array}{c}s.t.\sum\limits_{j=1}^{\overline{n} }\overline{{x }_{ij}}{\gamma }_{j}+{s}^{-}=\theta {x}_{ip}, i=1\cdots m\end{array}$$

3 $$\begin{array}{c}\sum\limits_{j=1}^{\overline{n} }d\overline{{y }_{ij}}{\gamma }_{j}-{s}^{+}={y}_{rp}, r=1\cdots s\end{array}$$

4 $$\begin{array}{c}\sum\limits_{j=1}^{\overline{n}}{\gamma }_{j}=1\end{array}$$

$$\begin{array}{c}{\gamma }_{j}\ge 0,j=\mathrm{1,2},\cdots n,{s}^{+}\ge 0,{s}^{-}\ge 0\#\end{array}$$

where $\theta$ is the relative efficiency value of the evaluated DUM, ${s}^{-}$ and ${s}^{+}$ are slack variables, the constant *d* is the movement factor that reflects the dynamic adjustment of the system, $\varepsilon$ is the non-Archimedean infinitesimal, and ${\gamma }_{jt}(j=\mathrm{1,2},\cdots n)$ is the weight coefficient.

In the second stage, conduct stochastic frontier analysis model (SFA) (Aigner et al. (1977)). We set the slack of input as the explained variable and the environmental factors as the explanatory variables; SFA is used to analyze the influence of environmental factors on the decision-making unit. The SFA model is shown in Eq. (5).

5 $${S}_{ij}={f}^{i}\left({z}_{j};{\beta }^{i}\right)+{v}_{ij}+{u}_{ij}$$

${S}_{ij}$ represents the slack variable of the ${i}_{th}$ input of the ${j}_{th}$ decision-making unit. ${z}_{j}$ are environmental variables, and ${\beta }^{i}$ are parameters to be estimated. ${v}_{ij}\sim N\left(0,{\sigma }_{ui}^{2}\right)$ are random interference, ${u}_{ij}\sim {N}^{+}\left(\mu ,{\sigma }_{ui}^{2}\right)$ are invalid management, and they are independent of each other.

For making each DUM in the same natural state, to measure and calculate the efficiency value that purely reflects the management level of each DUM, we adjust the input items of each DUM according to the calculation results $\left(\widehat{{\beta }^{i}},\widehat{{\sigma }_{ui}^{2}}\right)$ of the SFA regression model. At the same time, in order to ensure that the adjusted inputs are positive, as shown in Eq. (3), we use the worst external environment and maximum random interference as the benchmark and make adjustments by increasing the input of other DUMs; ${x}_{ij}$ are actual inputs, and ${x}_{ij}^{A}$ are adjusted inputs.

6 $${x}_{ij}^{A}={x}_{ij}+\left[{max}_{j}\left({z}_{j};{\beta }^{t}\right)-{z}_{j}{\beta }^{t}\right]+\left[{max}_{j}\left({v}_{ij}\right)-{v}_{ij}\right]$$

In the third stage, run DEA model again. The original inputs are replaced by the adjusted inputs from step 2, and the outputs remain unchanged. The traditional DEA model is run again to get the efficiency without the influence of environmental factors and random errors, which can more accurately reflect China’s regional green total factor productivity. According to the adjusted input value ${x}_{ij}^{A}$ of each DUM, we run the DEA model again to calculate the new efficiency value. After removing the influence of random interference and external environmental factors, the adjusted efficiency value can better reflect the actual efficiency level. When the efficiency value $\uptheta =1$, the DUM is effective; otherwise, it is non-effective. For non-effective DUM, we can calculate the input redundancy $\Delta {X}_{pt}=\left(1-\theta \right){X}_{pt}-{S}^{-}$ and the insufficient output $\Delta {Y}_{pt}={S}^{+}$ by the projection analysis based on the front surface.

We first use panel regression method to test the total impact of FDI on GTFP.

7 $$\begin{array}{c}{GTFP}_{it}={\beta }_{0}+{\beta }_{1}{FDI}_{it}+{\beta }_{2}{c}_{it}+{\varepsilon }_{it}\end{array}$$

Secondly, the mediating effect test is conducted to examine the channels that FDI affects GTFP.

8 $$\begin{array}{c}{GTFP}_{it}={\beta }_{0}+{\beta }_{1}{X}_{it}+{\beta }_{2}{c}_{it}+{\varepsilon }_{it}\end{array}$$

9 $$\begin{array}{c}{GTFP}_{it}={\beta }_{0}+{\beta }_{1}{FDI}_{it}+{\beta }_{2}{X}_{it} +{\beta }_{3}{c}_{it}+{\varepsilon }_{it}\end{array}$$

${GTFP}_{it}$ is the green total factor productivity of the ${i}_{th}$ region in the $t$ year. ${FDI}_{it}$ is the core explanatory variable of this paper and refers to foreign direct investment. $X$ includes three variables: innovation level, R&D investment level, and human capital accumulation level. The interaction term is mainly used to investigate the mediation effect of the research hypothesis. To prevent endogeneity problems caused by missing variables, we control a set of variables influencing GTFP. ${\varepsilon }_{it}$ is a random error.

In order to test the nonlinear impact of FDI on GTFP under different levels of marketization and environmental regulation, While further discussing the heterogeneous influence of FDI on GTFP under different levels of innovation, R&D investment, and human capital accumulation, we adopt the panel threshold regression model proposed by Hansen (1999). The advantage of this model is that it not only estimates the threshold value, but also tests the significance of the endogenous “threshold effect.” In this paper, environmental regulation level, marketization level, innovation level, R&D investment level, and human capital accumulation level are taken as threshold variables, respectively. The threshold panel model is the following:

10 $$\begin{array}{c}{GTFP}_{it}={\beta }_{0}+{\beta }_{1}{FDI}_{it}+{\beta }_{2}{FDI}_{it}I\left({Z}_{it}<\delta \right)+{\beta }_{3}{c}_{it}+{\varepsilon }_{it}\end{array}$$

${GTFP}_{it}$ is the same as shown above. ${Z}_{it}$ includes environmental regulation level, marketization level, innovation level, R&D investment level, and human capital accumulation level, which were used as the threshold variables of shadow GTFP, respectively. $\mathrm{I}\left(\bullet \right)$ is indicative coefficient; $\updelta$ is the calculated threshold value; different threshold variables correspond to a different threshold value; ${\beta }_{2}$ is the threshold regression coefficient of the core variable ${FDI}_{it}I$.

### Variables

#### The explained variable: GTFP

The explained variable is GTFP, measured by a three-stage DEA. In this paper, we select total energy consumption, material capital stock, and the number of employees as input indicators. We also select GDP as the expected output indicator and carbon dioxide emissions as the undesired output indicator. Considering the second stage, we need to eliminate those facts that affect the efficiency of GTFP and cannot be changed in a short time, including two key factors for full-time equivalent (FTE) of R&D and full-time equivalent (FTE) of R&D personal. GTFP is also affected by the large development gap between provinces in China, particularly the economic gap; we select the secondary industry’s proportion in GDP and the full-time equivalent (FTE) of R&D as environmental factors (Table 1).

**Table 1:** Description of variables

Variable type	Variable name	Symbol	Variable description	Unit	Mean	Standard deviation
Input variable	Energy consumption	EC	Total energy consumption	10,000 tons of standard coal	10,779.18	7702.26
	Material capital	MC	Physical capital stock	(people/10,000 yuan) (price in 2000)	17.45	58.88
	Labor input	Lab	Number of employed persons in urban	Ten thousand people	780.15	633.40
Output system	Economic development	Eco	*GDP*	Yuan (price in 2000)	9293.96	9305.57
	Carbon emission	Co^2^	Carbon dioxide emissions	Ten thousand tons	27,513.56	21,524.37
Environmental factor	Industrial development	Ind	The proportion of the secondary industry in GDP	%	46.44	7.78
	R&D investment level	Rd	The full-time equivalent of R&D personnel	Person-year	73,569.85	90,715.93

#### Core explanatory variable: FDI

FDI is measured by the ratio of actual utilization of foreign capital to GDP.

#### Control variables

(1) *Economic development level* is measured by per capita GDP; (2) *energy structure*, measured by the proportion of thermal power generation in total power generation; (3) infrastructure construction, measured by per capita urban road area; (4) *population quantity*, measured by the total population; (5) *urbanization rate*, measured by the proportion of the urban population in the total population; (6) *physical capital accumulation level*, measured by the stock of physical capital; and (7) *investment level*, measured by the fixed assets of the whole society total investment measurement.

#### Mediated variables and threshold variables

(1) *Innovation level* is measured by the number of domestic patent applications granted per capita; (2) *R&D investment level* is measured by the full-time equivalent of R&D personnel; (3) *human capital accumulation level* is measured by actual labor human capital. The policy regulatory factors are represented by *environmental regulation (ER)* and *marketization degree (Market)*; *ER* is measured by the level of regional environmental regulation by the proportion of emissions tax levied in GDP, while *market* is measured by the marketization index.

### Data source

Considering available data, we exclude the data from Tibet, Hong Kong, Macau, and Taiwan. The panel datasets were constructed by 30 provinces from 2000 to 2016 year. The primary data is calculated from the China Urban Statistical Yearbook and China’s Energy Statistical Yearbook. The worth mentioning is the energy consumption data from the statistical yearbooks of provinces, the marketization index measured from the Report with China’s Marketization Index by Wang et al. (2019), and the human capital accumulation data which comes from the actual labor human capital of each province calculated by the CHLR project.^3^ The index of carbon dioxide emissions collects from the eight types of energy consumption, including diesel consumption, coke consumption, coal consumption, kerosene consumption, gasoline consumption, fuel oil consumption, crude oil consumption, and natural gas consumption. Then evaluation the coefficient of energy conversion to the carbon. The inter-provincial material capital stock is calculated based on the relevant data and methods of Zhang (2008), the measuring equation as ${K}_{it}={K}_{it-1}\left(1-{\updelta }_{\mathrm{it}}\right)+{I}_{it}$. Table 2 shows the descriptive statistics of variables.

**Table 2:** Descriptive statistics of variables

Variable name	Variable name	Symbol	Variable description	Unit	Mean	Standard deviation
*Explained variable*	Green total factor productivity	*GTFP*	Efficiency after removing environmental interference factors	–	0.89	0.15
*Core explanatory variable*	Foreign direct investment	*FDI*	Total foreign investment/gdp	%	0.434	0.542
*Control variables*	The level of economic development	*pgdp*	GDP per capita	Yuan/person	49.382	35.178
	Clean energy utilization	*ce*	Proportion of thermal power generation in total power generation	%	0.78	0.224
	Infrastructure construction level	*inc*	Urban road area per capital	Square meter	12.069	4.336
	Population size	*pop*	population	Ten thousand people	2498.416	1670.947
	Urbanization rate	*urban*	The proportion of urban population in total population	%	48.175	15.307
	Social investment level	*inv*	Total investment in fixed assets of the whole society	Ten thousand yuan	116,286.7	139,419.6
	Material capital	*k*	Material capital accumulation level (price in 2000)	100 million yuan	17.448	58.88
*Mediated variables and threshold variables*	Innovation level	*inn*	Material capital accumulation level (price in 2000)	Pieces/10,000 people	4.233	7.175
	R&D investment level	*Rd*	Full-time equivalent of R&D personnel	Person-year	73,569.85	90,715.93
	Human capital level	*rlab*	Labor force human capital	–	3598.689	2742.471
	Environmental regulation	*er*	The percentage of emissions tax in GDP	%	0.2418638	0.3610417
	Marketization level	*market*	Marketization index	–	6.642	2.083
*Other variables*	Energy consumption per unit of GDP	*enc*	Total energy consumption/gdp	Ton of standard coal/yuan (price in 2000)	1.99942	2.000756
	Industrial structure	*ins*	The proportion of the secondary industry in GDP	%	46.437	7.778
	Economic scale	*ec*	Gross national product	Yuan (price in 2000)	9293.956	9305.565
	emission intensity	*ei*	Exhaust emissions (sulfur dioxide; nitrogen dioxide; carbon dioxide)/gdp	TONE/ten thousand yuan(Price in 2000)	289.8263	332.4328

Note: The marketization index from the China’s Marketization Index Report by Provinces.

## Results

### Baseline results

Considering the estimation bias caused by regional heterogeneity and time factors, the dynamic panel regression model with the two-way fixed effect is used to estimate the parameters; the estimation results are shown in columns 1–3. The basic estimation result is shown in column 1, and columns 2 and 3 perform the robustness test by replacing explained variables. In addition, considering traditional estimation methods is powerless in controlling potential endogenous problems, refer to Blundell et al. (2000); the system GMM method can be used to estimate the dynamic panel model; the estimation results are reported in columns 4–6. As the results shown in column 1, the impact of FDI on regional GTFP is significantly positive at the 5% level with controlling series fixed effects, indicating that FDI will promote GTFP in China. As mentioned above, existing researches have been controversial on the relationship between FDI and GTFP, and the empirical results of this paper prove the positive role of FDI in promoting GTFP. Although the purpose of foreign investment entering China is to seek relatively cheap production costs and relatively loose environmental regulations and transfer industries with high pollution and high energy consumption from home country to China, however, the more standardized production process and technology brought by FDI can also effectively improve resources utilization and promote the environment. We perform a robustness test by replacing GTFP with energy consumption per unit of GDP(*ENC*) and emission intensity (*EI*), respectively; the estimation results are shown in columns 2–3. The coefficients of *ENC* and *EI* are significantly negative at the 5% level, which once again proves the “pollution halo effect” of FDI in China. The columns 4–6 list the estimation results of the GMM model; we use the FDI term with a lag period as the instrumental variable. Although the regression results were not significant after replacing GTFP with ENC and EI, the direction of the coefficients was consistent (Table 3).

**Table 3:** Regression

	Two-way mixed effects model			GMM model		
	(1)	(2)	(3)	(4)	(5)	(6)
	GTFP	ENC	EI	GTFP	ENC	EI
FDI	0.0226** (2.37)	− 0.363** (− 2.17)	− 69.99** (− 2.13)	0.0262* (1.91)	− 0.207 (− 1.10)	− 31.54 (− 0.92)
pgdp	0.00159*** (4.91)	− 0.0359*** (− 6.33)	− 5.923*** (− 5.81)	0.00225*** (4.21)	− 0.0459*** (− 5.48)	− 5.117*** (− 3.42)
es	0.00166 (0.03)	− 2.303** (− 2.22)	− 373.4 (− 1.61)	0.254* (1.88)	− 6.083*** (− 3.12)	− 295.0 (− 0.79)
Inc	0.00714*** (4.73)	− 0.192*** (− 7.13)	− 40.04*** (− 8.25)	0.00987*** (4.04)	− 0.239*** (− 6.23)	− 39.47*** (− 5.67)
pop	-0.0000172 (-0.91)	0.000562* − 1.71	0.0492 − 0.83	− 0.0000161 (− 0.55)	− 0.0000209 (− 0.05)	0.0248 (0.31)
urban	0.000428 (0.89	− 0.0281*** (− 3.44)	− 1.883 (− 1.20)	0.000878 (1.18)	− 0.00522 (− 0.46)	− 2.369 (− 1.06)
inv	− 0.000000104*** (− 4.56)	0.00000176*** (4.32)	0.000377*** (5.08)	− 0.000000193*** (− 4.12)	0.00000315*** (4.48)	0.000441*** (3.54)
k	0.00011 (0.76)	0.00449* (1.75)	1.081** (2.25)	0.0000184 (0.08)	0.00767** (2.36)	1.358** (2.40)
Province, year	Control	Control	Control	Control	Control	Control
_cons	0.727*** (9.9)	9.419*** (7.29)	1555.8*** (5.43)	0.503*** (3.96)	11.17*** (6.12)	1168.6*** (3.43)
*N*	451	480	300	451	480	300

*p < 0.10, **p < 0.05, ***p < 0.01; standard errors in parentheses.

### Mechanism analysis

In order to investigate the underlying channels through which FDI may affect GTFP, we introduce the cross-terms of innovation level (*Inn*), R&D investment level (*Rd*), and human capital level (*Rlab*) and FDI, respectively, and test the coefficients. The estimation results are shown in Table 4.

**Table 4:** Estimated results

	(1)	(2)	(3)	(4)	(5)	(6)
	GTFP	GTFP	GTFP	GTFP	GTFP	GTFP
FDI		0.0288*** (2.87)		0.0234** (2.46)		0.0372*** (− 3.47)
Inn	− 6.484** (-3.16)	− 3.253 (− 1.14)				
Inn *FDI		− 4.036* (− 1.65)				
Rd			− 0.000000348*** (− 3.70)	− 0.000000109 (− 0.71)		
Rd*FDI				− 0.000000403* (− 1.82)		
Rlab					− 0.0000214** (− 2.88)	− 0.0000149* (− 1.88)
Rlab*FDI						− 0.0000199** (− 2.50)
Control variables	Yes	Yes	Yes	Yes	Yes	Yes
Province, year	Control	Control	Control	Control	Control	Control
_cons		0.776*** (11.58)		0.751*** (10.89)		0.769*** (10.2)
*N*		451		451		451

*p < 0.10, **p < 0.05, ***p < 0.01; standard errors in parentheses.

The estimation results reported in columns 2 and 4 show that the interaction terms of FDI and innovation level and R&D investment level have a significant inhibitory effect on GTFP at the 1% and 5% level, respectively. These results indicate that innovation and technology spillover brought by foreign direct investment does not improve regional green total factor productivity. As mentioned above, the positive effect of innovation and technology spillover requires certain conditions that local enterprises should have corresponding absorption and transformation capacity. If technology is only introduced in single-direction dissemination without improving the absorptive capacity of local industries, the technology introduction brought by FDI will not improve the economic growth rate of the host country (Keller 1998; Suyanto et al. 2010). Although China has actively introduced foreign investment since the 1980s, the technology absorption capacity of local enterprises is relatively weaker, especially for a large number of small and medium-sized companies, which cannot find ways to move away from relying solely on technology import. This phenomenon is associated with the heterogeneous FDI, in the context of the tournament-based organization; local governments of China tend to do their best to attract foreign investment to get the better of an adversary, so the quantity of FDI is more important than the quality of FDI for them. In addition, the goal of a number of multinational enterprises and investments entering to China is to avoid environmental costs and use an abundant supply of cheap labor and resources. These types of FDI without bring positive demonstration effect and promote GFTP.

The estimation results reported in column 6 show that the interaction term between *FDI* and *Rlab* has a significant negative effect on GTFP with at 5% level, indicating that FDI does not promote regional GTFP in China through the human capital spillover effect. Technological progress is inseparable from improving the quality of the population; for developing countries, the level of human capital directly affects the ability to imitate and absorb the advanced technology of developed countries and determines whether developing countries can achieve economic catch-up with developed countries. These results reveal that multinational enterprises may not help local enterprises to train technicians, which participate in GVC as OEM (Haskel et al. 2007); local Chinese employees do not take important positions such as R&D and senior management in enterprises.

In general, the human capital spillover effect has a better impact on GTFP. It is worth noting that China has a “polluting halo” effect, but the innovation spillover effect, technology spillover effect, and human capital spillover effect all restricted the improvement of GTFP, with the human capital spillover effect having the slightest inhibitory effect on GTFP. The following are some plausible explanations. First, many Chinese SMEs have depended on obtaining technological patents and importing equipment to grow their manufacturing scale since China’s entrance to the WTO. On the other hand, local SMEs rely on cheaper labor and energy costs to keep costs down. This development model overlooks the adoption and transformation of sophisticated technologies and the buildup of human capital, resulting in a managerial and technological gap. This route dependency limits FDI’s ability to boost China’s GTFP. Second, the enrollment rate and the average years of schooling have increased significantly with increasing financial investment in primary education. Moreover, influenced by traditional culture, Chinese families always invested a large part of their wealth in education. Chinese middle-class parents generally actively support their children to enter university for further studies. The massive investment in education by Chinese families has effectively supplemented the regional gap of public education investment. With the support of the government and family, the quality of the labor force is significantly improved. Therefore, although the role of FDI in the training of local talents is limited, the general improvement in the quality of China’s labor force has effectively made up for this deficiency.

### Estimation results of panel threshold regression

We use the panel threshold regression model to further investigate the nonlinear impact of FDI on GTFP; estimation results are reported in Table 5. In order to select the appropriate number of thresholds and threshold values, it is necessary to conduct threshold tests on variables respectively. The results reported in Table 5 show that marketization level (*Market*), innovation level (*Inn*), R&D investment level (*Rd*), and human capital level (*Rlab*) pass the double threshold test at the 1% significance level, and environmental regulation level (*ER*) passes the double threshold test at the 5% significance level. The threshold values obtained after the threshold effect test are (3.37, 5.82), (2.182, 3.095), (0.001, 0.002), (4187.8, 116,842), and (466.564, 8274.15) respectively.

**Table 5:** Threshold effect test

	*Market*			*Inn*			*Rd*			*Rlab*			*ER*		
	*F* value	*p* value	10%	*F* value	*p* value	10%	*F* value	*p* value	10%	*F* value	*p* value	10%	*F* value	*p* value	10%
Single	80.102	0.000	29.154	18.856	0.020	8.550	52.413	0.000	6.216	80.932	0.000	37.988	17.238	0.02	9.905
Double	19.266	0.000	4.552	13.650	0.017	7.780	26.747	0.000	-20.286	28.703	0.000	2.967	3.855	0.057	2.942

The* p* value is the result obtained after 300 simulations using the Bootstrap method.

The estimation results in columns 1–3 (Table 6) show that when the marketization level is low, the coefficient of FDI is significantly negative, and as the marketization level breaks through the threshold value, the FDI coefficient becomes significantly positive. It indicates that in the early stage of economic openness with the high level of market segmentation and local protectionism, local governments aim to attract FDI and lose environmental standards. Moreover, due to the limited inter-regional factor flow, the technology and human capital spillover effect brought by foreign investment just have a partially positive influence on the improvement of regional productivity. But with the increasing marketization level, the environmental benefits brought by FDI will quickly become apparent, relating to the local governments voluntarily restricting the standards to reject the FDI with potential environmental risks and strengthening regional cooperation. The estimation results in columns 4–7 show that even when the environmental regulation level is low, the coefficient of FDI is still significantly positive, and with the improvement of environmental regulation level, the coefficient of FDI keeps increasing. It indicates that if local governments start environmental governance, it is difficult for FDI with pollution risks to enter China, while clean FDI can effectively improve the clean production capacity of local enterprises. In these circumstances, foreign enterprises should strictly implement unified environmental standards and apply advanced clean technology in the production process. But if environmental regulation is too strict for multinational firms to get profit, it will withdraw from the market and find the other destination with lower environmental costs, and local firms will lose the opportunity to learn by doing. The crowding-out effects can explain the negative impacts of FDI leading to strict environmental regulation on regional GTFP.

**Table 6:** Threshold regression

	*Market*			*ER*			
	< 3.37	(3.37,5.82)	≥ 5.82	< 2.182	(2.182, 3.095)	(3.09, 5.786)	≥ 5.786
FDI*I	− 0.161*** (− 7.47)	− 0.156*** (− 7.36)	0.195* (1.76)	0.0438*** (2.84)	0.0660* (1.84)	0.00755 (0.24)	− 0.0251 (− 1.56)
Control variables	Yes	Yes	Yes	Yes	Yes	Yes	Yes
Constant	0.470***	0.526***	0.282***	0.507***	0.504***	0.504***	0.342***
	(12.60)	(13.51)	(6.32)	(13.27)	(13.22)	(13.22)	(8.35)
N	451	451	451	451	451	451	451

*p < 0.10, **p < 0.05, ***p < 0.01; standard errors in parentheses.

Table 7 reports the further analysis of the innovation, technology, and human capital spillover effect of FDI on GTFP. The estimation results in columns 1–3 show that when the independent innovation level of local enterprises is low, FDI has a significant negative impact on regional GTFP. However, with the local innovation level increasing, the environmental benefits of FDI will gradually emerge. It indicates that only when the local firms have the capacity to abort advanced technology, the technology spillover effect of FDI can play a positive role in promoting GTFP. On the contrary, if local firms cannot be able to digest the advanced technology with path dependence of technology import, FDI has negative influence on the development of indigenous innovation ability. The regression with R&D investment level and human capital accumulation level as threshold variables has similar results.

**Table 7:** Threshold regression

	*Inn*			*Rd*			*Rlab*		
	< 0.001	(0.001, 0.002)	≥ 0.002	< 4187.8	(4187.8, 116,842)	≥ 116,842	< 466.564	(466.564, 8274.15)	≥ 8274.15
FDI*I	− 0.0869*** (− 4.04)	0.0782*** (3.53)	0.0899*** (3.72)	− 0.0612*** (− 3.59)	0.0931*** (4.32)	0.0607** (2.55)	− 0.0927*** (− 4.83)	0.155*** (2.99)	0.245*** (5.11)
Control variables	Yes	Yes	Yes	Yes	Yes	Yes	Yes	Yes	Yes
Constant	0.643*** (16.84)	0.636*** (16.86)	0.386*** (10.41)	0.527*** (12.93)	0.520*** (13.09)	0.398*** (10.68)	0.429*** (11.32)	0.411*** (11.12)	0.289*** (7.72)
*N*	451	451	451	451	451	451	451	451	451

*p < 0.10, **p < 0.05, ***p < 0.01; standard errors in parentheses

### Heterogeneity analysis

Table 8 reports the group regression estimation results divided by economic region of China, showing that FDI has heterogeneous influences on GTFP in different economic regions. Columns 1 and 2 report the estimation results of eastern and central regions. Although the coefficients are not significant, it can partially explain that FDI has a positive impact on GTFP in eastern and central regions. Meanwhile FDI plays a significant negative impact on GTFP in western and northeastern regions. The results indicate that the regional heterogeneous effects brought by FDI on GTFP relating to regional development level and resource endowment, which has heterogeneity and needs further analysis.

**Table 8:** Regional heterogeneity analysis

	Eastern	Central	Western	Northeastern
	GTFP	GTFP	GTFP	GTFP
FDI	0.0148 (1.19)	0.159 (1.11)	− 0.221*** (− 2.85)	− 0.246** (− 2.27)
Control variables	Yes	Yes	Yes	Yes
_cons	0.862*** (5.35)	0.825*** (6.43)	0.785*** (7.02)	1.024*** (3.04)
*N*	136	105	165	45

*p < 0.10, **p < 0.05, ***p < 0.01; standard errors in parentheses. According to the division method of Chinese administrative regions, the provinces are divided into Northeastern, Eastern, Central, and Western China. The Northeastern provinces include Jilin, Liaoning, and Heilongjiang. The Eastern provinces include Hebei, Beijing, Tianjin, Shandong, Jiangsu, Shanghai, Zhejiang, Fujian, Guangdong, and Hainan. Central provinces include Henan, Hubei, Hunan, Anhui, Jiangxi, and Shanxi. The Western provinces include Chongqing, Sichuan, Guizhou, Yunnan, Guangxi, Shaanxi, Gansu, Qinghai, Ningxia, Xinjiang, and Inner Mongolia.

According to the estimation results in columns 2 and 3 of Table 9, we find that in regions with large economic scale (divided by the median of economic scale), FDI has no significant positive effect on GTFP, while in regions with small economic development scale, FDI can significantly improve GTFP. The possible reason is that, compared with the regions with smaller economic scale, the regions with larger economic scale have entered the development stage of diminishing marginal utility with the investment which has tended to saturation, formed mature development paths, so the environmental benefits brought by FDI are limited. The estimation results in columns 4 and 5 show that the industrial structure level (divided according to the median value of industrial structure level) has a significant impact on improving the environmental benefits of FDI. In regions with more advanced industrial structure, the government sets a higher standard for investors and prefers clean FDI with the development of an environment-friendly industrial structure. At the same time, local enterprises can adopt foreign advanced technology and clean production model to improve production efficiency and reduce pollution.

**Table 9:** Regional heterogeneity analysis

	Regions with larger economies	Regions with smaller economies	Regions with high level of industrial structure	Regions with low level of industrial structure
	GTFP	GTFP	GTFP	GTFP
FDI	0.0111 − 0.61	0.0286** − 2.41	0.0625*** − 5.45	− 0.00284 (− 0.09)
Control variables	Yes	Yes	Yes	Yes
_cons	1.050*** − 11.79	0.589*** − 5.45	0.809*** − 6.83	0.839*** − 8.6
*N*	167	284	236	215

*p < 0.10, **p < 0.05, ***p < 0.01; standard errors in parentheses. We divided regions into high-low level based on the median.

## Conclusions and policy implications

The relationship between environmental protection and economic growth has been a hot topic in the past decades; however, energy consumption has risen with countries’ industrialization since the industrial revolution. As the world’s biggest energy consumer, China should play an important role in fighting global warming and reducing carbon emissions. The community of shared future for mankind proposed by the Chinese government focus on ecological crisis response with mobilizing international support. Especially, China tries to build an environment-friendly society; the clean production is an essential means to achieve this goal. This paper enriches the existing literature by discovering the potential effects of FDI on the green total factor productivity of developing countries (especially the understudied region of China) and testing the threshold effects of regional marketization and environment regulation level and the absorptive capacity of local firms to analyze to what extent regional green total factor productivity benefit from foreign investment.

First of all, the results show that FDI has a positive impact on regional GTFP in China. Based on these results, we verify the pollution halo effect of FDI and argue that the important role of FDI plays in promoting clean production and environmental protection in China. Second, through the innovation spillover effect, technology spillover effect, and human capital spillover effect, FDI has heterogeneous impacts on GTFP. Considering the absorption capacity of local firms, only when the innovation level, R&D investment level, and human capital level break through the threshold values, the spillover effects of FDI can have a positive impact on regional green total factor productivity. On contrary, the crowing effects of FDI may lead to a pollution paradise phenomenon. There is a nonlinear relationship between FDI and GTFP with different levels of marketization and environmental regulation. With increasing improvement of marketization level, the environmental benefits of FDI decrease first and then increase, indicating that the free flow of factors and regional cooperation is helpful to weaken the crowding effects of FDI. However, results also show that there is a reciprocal U kink of regional environment regulation level. once local governments start environmental regulation, FDI has a positive impact on regional green total factor productivity. However, if the environmental regulation level is close to a turning point, foreign capital will shift to other destinations for lower environmental costs. Generally speaking, environmental regulation is beneficial to regional sustainable development. Last, the impacts of FDI on GTFP represent the characteristics of regional heterogeneity. Western region and northeast region are the “pollution paradise” in China, and the environmental benefits of FDI vary in regions with different economic scales and industrial structure levels.

Based on the research results, this paper gives the following policy recommendations.

1. The Chinese government should continue to encourage foreign direct investment, but it is necessary to formulate practical introduction environmental standards and environmental policies to promote cleaner production in China. First, local governments should formulate formal environmental regulatory policies that meet the local human capital level and technological level considering local characteristics. Second, local governments should actively undertake international high-tech enterprises with low emissions, promote the development of green industry, and continuously improve the level of green economy development. Third, local governments need to reasonably subsidize the green technology upgrades of companies, establish emission trading systems to achieve effective resource allocation, and improve the efficiency of green innovation factor allocation.
2. The government needs to improve various supporting systems for the promotion of human capital. First, considering labor market demand, the government needs to pay more attention to vocational education *t* and raise the skilled workers’ social position. Second, establish a guarantee mechanism for the free flow of human capital. The government should actively promote the reform of the household registration system and eliminate the institutional obstacles in labor allocation. For example, local governments can provide the free service of releasing labor market information to match supply and demand in the labor market. Local governments also should support the market intermediary’s development of human resource services and labor, encouraging the market power to play a decisive role in personnel allocation.

The government should pay attention to intellectual property protection and focus on cultivating the independent innovation capacity of local enterprises. In the past, the poor protection of intellectual property rights led to serious piracy, and it has greatly damaged original enterprises that have invested much money in research and development. It is necessary to protect the researchers’ intellectual property rights and originators’ economic rights to stimulate innovation enthusiasm. For example, in 2019, Shanghai pledged to improve its business environment. Apart from improving government efficiency, one of the essential points is forming a standardized market environment and protecting intellectual property rights. The continuous recognition of technological innovation achievements by the market can increase the investment in scientific research and guide scientific research institutions to meet the market demand to transform technological achievements actively.

## Data Availability

The datasets used and/or analyzed during the current study are available from the corresponding author on reasonable request.

## Footnotes

1 Data source: 《BP Statistical Yearbook of World Energy (2019)》

2 http://www.xinhuanet.com//politics/19cpcnc/2017-10/18/c_1121820882.htm

3 http://humancapital.cufe.edu.cn/rlzbzsxm/zgrlzbzsxm2020.htm
